# Evaluation of retention of pit and fissure sealants placed with and without air abrasion pretreatment in 6-8 year old children – An *in vivo* study

**DOI:** 10.4317/jced.53259

**Published:** 2017-02-01

**Authors:** Urvashi Bhushan, Mridula Goswami

**Affiliations:** 1 BDS, Postgraduate student in Department of Pedodontics and Preventive Dentistry at Maulana Azad Institute of Dental Sciences, New Delhi; 2 BDS, MDS, Head of the Department & Professor, Department of Pedodontics and Preventive Dentistry at Maulana Azad Institute of Dental Sciences, New Delhi

## Abstract

**Background:**

The success of pit and fissure sealants is directly related to their retention. The purpose of this study was to assess and compare the retention of pit and fissure sealants placed using acid etch alone and a combination of air abrasion and acid etch techniques.

**Material and Methods:**

50 subjects aged 6-8 years were included in the study. Primary second molars and permanent first molars were sealed in all four quadrants using split mouth design. The right maxillary and mandibular molars (Group A) were treated by acid etching alone while the left maxillary and mandibular molars (Group B) were pretreated with air abrasion followed by acid etching before application of pit and fissure sealant. Retention of sealants was checked using Simonsen’s criteria of sealant retention after three and six months of sealant application.

**Results:**

There was no significant difference in retention of sealants in Group A and Group B (*p*<0.05) after three and six months follow up. The difference in sealant retention in primary and permanent molars was not significant (*p*>0.05). Maxillary molars showed superior retention compared to mandibular molars, which was statistically significant at both three and six months (*p*<0.05).

**Conclusions:**

Combining air abrasion pretreatment with subsequent acid etching did not result in statistically significant difference in sealant retention compared to acid etching alone in both primary and permanent molars after 3 and 6 months follow up. An additional air abrasion pretreatment step can be avoided in pediatric patients when placing sealants and the procedure can be completed faster with better behavior management using acid etching alone.

** Key words:**Pit and fissure sealant, acid etching, air abrasion.

## Introduction

Prevention of oral diseases is preferable to treatment and is the key method of achieving cost effectiveness for oral health improvement programs. Prevention results in less pain and trauma to the patient and reduces the need for highly trained professional personnel. Various preventive strategies for dental caries have been tried and are still being developed. The occlusal pits and fissures of posterior teeth are highly susceptible to caries because of the anatomy of pit and fissure surfaces, which favours stagnation of bacteria and substrates (1). Fissure sealing has been shown to be an evidence- based caries preventive method for protecting the occlusal surfaces against caries (2). Non- sealed teeth need to be restored approximately 50% more frequently compared to their sealed counterpart (3).

Sealants are effective caries preventive agents as long as they remain bonded to teeth (4). The different methods recommended to improve sealant retention include cleaning of the occlusal surface prior to sealant placement with hydrogen peroxide, pumice prophylaxis, air polishing, mechanical preparation of fissures and air abrasion. Acid etching is the evidence-based method for enamel preparation before fissure sealing (5). However, concern has been expressed that the traditional acid etch technique for sealant placement does not allow for complete cleaning of the pits and fissures prior to sealant placement (6). A new method for sealant application using air- abrasive technology is less technique sensitive and allows for further cleaning of the grooves prior to sealant placement. The abrasive particles used in air abrasion effectively remove organic plug material from the grooves and allow for deeper penetration of the sealant material into the grooves.

However, marginal leakage studies have shown that air abrasion alone is not as effective as air abrasion coupled with acid etching in preventing microleakage (7). An uncertainty for the best enamel surface pretreatment before sealant application still exists even after years of research studies. This study attempts to evaluate and compare the efficacy of two enamel surface pretreatment techniques - Acid etching alone or combination of air abrasion followed by acid etching technique.

## Material and Methods

The present study is a longitudinal experimental study carried out after obtaining prior approval from the Ethical Committee of the Institution. The study population comprised of 50 children aged 6-8 years who met the inclusion and exclusion criteria.

•Inclusion criteria: Absence of restorations or prior sealants on the teeth under study, absence of cavitated carious lesions, cooperative child patient, consent for treatment.

•Exclusion criteria: Medically compromised patients with history of respiratory disease, mentally and physically challenged patients. The details of the study procedure and the purpose of the study were explained to the parents and written Informed Consent was obtained from them.

Each child selected for the study received preventive treatment of application of pit and fissure sealants in the form of split mouth design. The right maxillary and mandibular primary 2nd molars and permanent 1st molars (Group A) were treated by acid etching alone while the left maxillary and mandibular primary 2nd molars and permanent 1st molars were pretreated with air abrasion followed by acid etching (Group B) before application of pit and fissure sealant.

The protocol for sealant placement using the acid etching technique (Group A) and acid etching with air abrasion pretreatment (Group B) was as follows:

1. ISOLATION OF TEETH

Isolation was achieved with the help of rubber dam for all four quadrants. After suitable selection of rubber dam clamps, teeth were isolated either arch wise or quadrant wise as per convenience and cooperation of the child.

2. ENAMEL SURFACE PRETREATMENT

(a) For Group A (Acid etch only): 37 % phosphoric acid solution was applied to primary second molar and permanent first molar occlusal pits and fissures with the help of applicator tip and left for 15 seconds for etching to occur. Teeth were subsequently rinsed with water for 20 seconds and then air-dried using three way syringe. Both primary and permanent molars were etched for 15 seconds.

(b) For Group B (Acid etch with air abrasion pretreatment): Air abrasive system with 50 micron alumina particles was used for 5 seconds at 5 mm distance (as per manufacturer’s instructions) from tooth surface with predetermined angle of 1380 (fixed nozzle in Standard model) and air pressure of 60-80 lbf/pol2, followed by etching as done for Group A.

3. PIT AND FISSURE SEALANT APPLICATION

A light curing, resin-based, color changing, unfilled pit and fissure sealant was applied to etched pits and fissures of occlusal surface using applicator tips. Sealant was cured with the light curing unit for 20 seconds as per manufacturer’s instructions. The sealant was pink when applied and on curing turned opaque white. After polymerization of pit and fissure sealant, rubber dam was removed and occlusion was checked with articulating paper. In case of high points, they were reduced using composite finishing burs. Patients were discharged and scheduled for recall visits at 3 and 6 months interval.

4. FOLLOW UP CLINICAL EVALUATION

Subjects were clinically evaluated after 3 and 6 months of sealant placement by study supervisor as a blinded outcome assessor. Follow up examinations were conducted in dental chair with the aid of mouth mirror. The criteria used for evaluation was according to Simonsen’s criteria (8) which is as follows :

-Completely retained- If some peripheral fissures were uncovered following sealant wear, but no ledges were visible.

-Partially retained- If, following either wear or material loss, part of a previously sealed pit/fissure was exposed.

-Missing- No trace of sealant is detectable.

After completing the data for 400 teeth in 50 children, they were followed up for 3 and 6 months in which 43 children reported. Data analysis was performed using Statistical Package for the Social Science-21 (SPSS-21). The data was analyzed on the basis of categorical scores given to retention of sealants using chi- square analysis. *P*-values < 0.05 were considered statistically significant.

## Results

**Figure 1:**
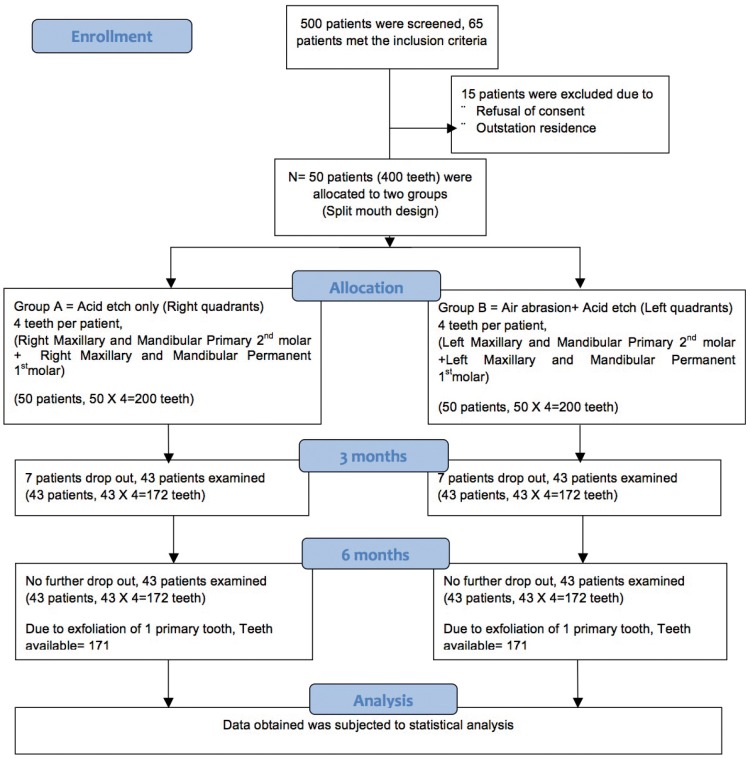
Schematic diagram showing sample design and follow up losses after 3 and 6 months.

A schematic diagram showing sample design is presented in figure 1. Out of 50 participants, there were seven drop outs and 43 reported for 3 and 6 months follow up. There was exfoliation of two primary teeth (one from each of the two Groups A and B) after 6 months. So number of teeth available for assessing sealant retention were 344 after 3 months and 342 after 6 months (Fig. 1).

I. Results of sealant retention comparing Acid etch and Acid etch with Air abrasion pretreatment after 3 months and 6 months are shown in  Table 1.

**Table 1:**
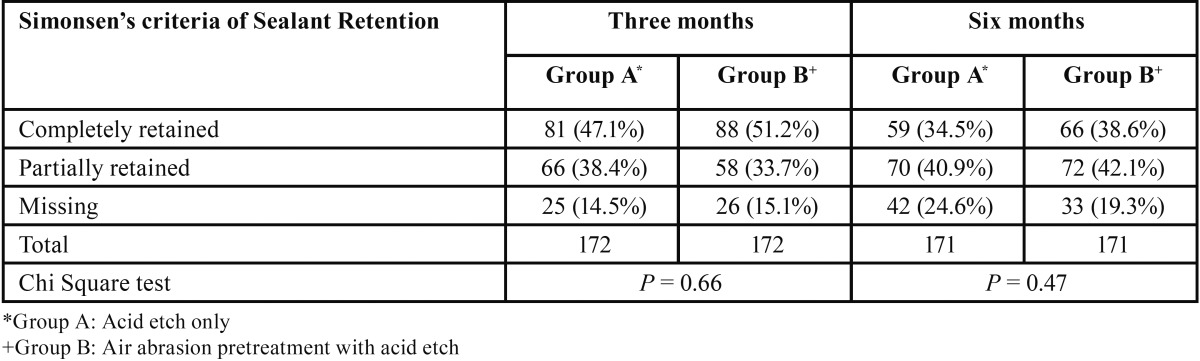
Distribution of sealant retention in Group A and Group B after 3 and 6 months.

The percentage of completely retained sealants was higher for Group B (51.2%) compared to Group A (47.1%) after 3 months follow up. However, the difference in sealant retention in two techniques was not found to be statistically significant (*P* > 0.05).

The percentage of completely retained sealants was higher in Group B (38.6%) compared to Group A (34.5%) after 6 months follow up. More number of sealants were missing in Group A (24.6%) than Group B (19.3%). However, the difference in sealant retention in two techniques was not found to be statistically significant (*P* > 0.05).

II. Results of sealant retention comparing Primary teeth (Maxillary + Mandibular) and Permanent teeth (Maxillary + Mandibular) after 3 and 6 months are shown in  Table 2.

**Table 2:**
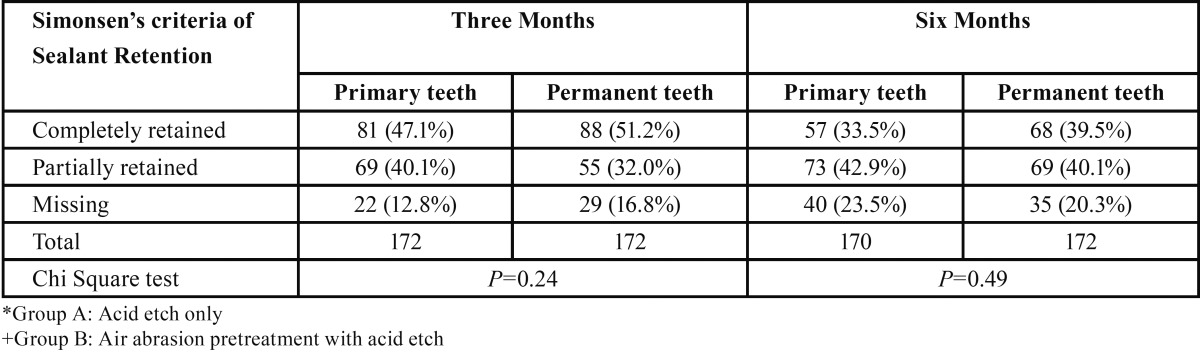
Distribution of sealant retention in primary teeth and permanent teeth after 3 and 6 months.

The percentage of completely retained sealants was found to be more for permanent first molars (51.2%) compared to primary second molars (47.1%) after 3 months follow up. However, the difference was not found to be statistically significant (*P*> 0.05).

The percentage of completely retained sealants was found to be more for permanent first molars (39.5%) compared to primary second molars (33.5%) after 6 months follow up. However, the difference was not found to be statistically significant (*P* > 0.05).

**Figure 2:**
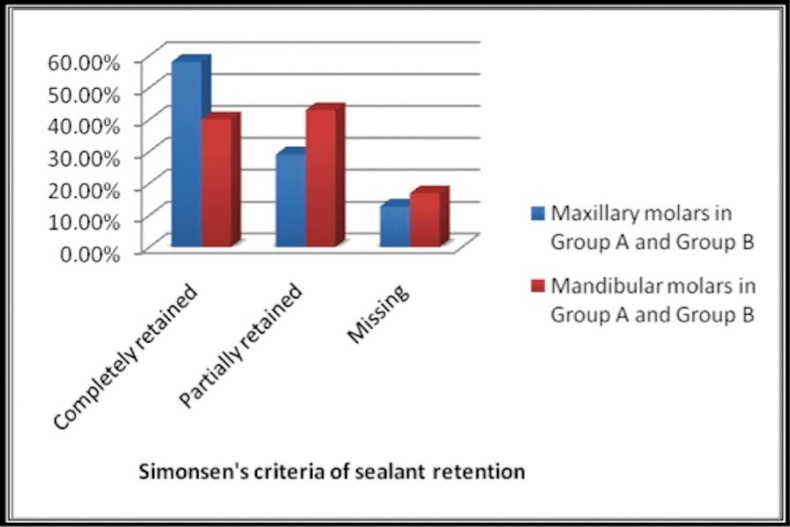
Sealant retention in maxillary and mandibular molars after 3 months.

**Figure 3:**
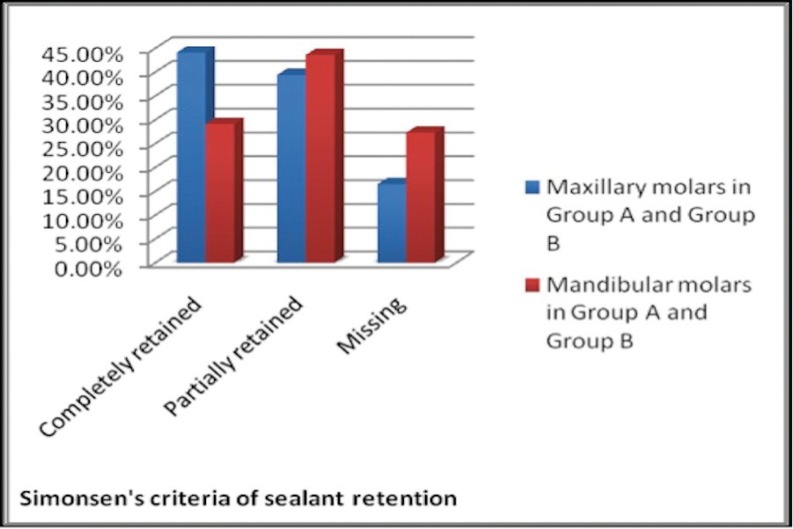
Sealant retention in maxillary and mandibular molars after 6 months.

III. Results of sealant retention comparing Maxillary molars (Primary + Permanent) with Mandibular molars (Primary + Permanent) after 3 months and 6 months are shown in figure 2 and 3 respectively.

The percentage of completely retained sealants was more for maxillary molars (58.1%) compared to mandibular molars (40.1%) after 3 months follow up. The difference was found to be statistically significant (*P*<0.05).

The percentage of completely retained sealants was more for maxillary molars (44.1%) compared to mandibular molars (29.1%) after 6 months follow up. The difference was found to be statistically significant (*P*<0.05).

## Discussion

The success of pit and fissure sealant depends on its long term retention on tooth surface. The prerequisite for retention is that the enamel surface be modified either with acid conditioning agent or some other technique such as air abrasion (9). The comparison of acid etch technique and air abrasion followed by acid etch technique on the retentiveness of fissure sealant was the aim of the study. This particular comparison was undertaken as concern has always been expressed in literature that the traditional acid etch technique for sealant placement does not allow for complete cleaning of the pit and fissures prior to the sealant placement (6). The retention of sealants with only acid etching is questionable. Several studies have shown some microleakage when sealants were placed by conventional method of acid etching (10,11).

It is known that primary and permanent teeth are different morphologically and histologically. Thus primary teeth can present differences in sealant retention after acid etching and air abrasion. Hence, both permanent and primary teeth were included in the study. It was also determined whether particular tooth location i.e maxillary teeth versus mandibular teeth is related to sealant loss or retention as there have been conflicting studies on it (12,13).

The study sample comprised of children between 6-8 years of age. Teeth chosen for sealant application were first permanent molars and second primary molars of all four quadrants in each subject. Children of this age group were chosen as first permanent molars erupt by the age of six years and show high incidence of caries soon after eruption. First permanent molars were chosen as their occlusal surface is most frequently attacked by dental caries (14). Second primary molars were included as no *in vivo* study has evaluated sealant retention on primary teeth comparing acid etch and combination of air abrasion and acid etch to the best of our knowledge. Occlusal surface of second primary molars is larger than first primary molars, enabling convenience in visual assessment of sealants because of broader occlusal surface.

A Split mouth design was used to avoid bias due to confounding factors such as different biting pressures, oral hygiene practices, dietary habits etc. and to ensure that similar conditions exist for both the techniques. Each participant acted as his own subject and control, so fewer participants were required to obtain same study power compared to parallel group design. All sealants were placed under rubber dam isolation, as rubber dam provides the most controllable isolation (15). In addition, it is impracticable to use air abrasion without using a rubber dam, as using air abrasion system creates alumina dust in the working area (16).

A concentration of 30-40% phosphoric acid etching provides enamel surfaces that have the most retentive appearance (17). In the present study, 37% phosphoric acid solution was used with an etching time of 15 seconds. Etching time of 15 seconds has been supported in literature for both primary teeth and permanent teeth (18). With the reduction of etching time, more enamel is preserved without affecting the clinical adhesion of the sealant. The air abrasive technology in dentistry has added a new potential method of pretreating teeth prior to placing sealants. Air abrasion units allow the clinician to focus a stream of aluminum oxide particles on a specific area of the tooth. Investigations on air abrasive techniques have suggested that this method may serve as an alternative to acid etching of enamel. A combination of air abrasion and phosphoric acid etch pre-treatment has been reported to create an enamel surface, whereby the bonded sealant material has demonstrated the highest shear bond strengths to intact enamel (19). In this study, Bio-Art Microblaster was used for air abrasion with alumina particles of 50 microns size. Alumina particles of size 50 microns were used as they abrade the tooth faster as compared to 27 microns alumina particles (20). A 5 mm distance was kept between the nozzle tip and tooth surface with predetermined angle of 1380 according to manufacturer’s instructions.

A resin based, fluoride releasing, unfilled and color changing sealant (Clinpro Sealant, 3M-ESPE) was used in the study. It has good fracture resistance and being a color changing sealant, it is easy to assess application before curing (21). Retention of sealants was evaluated using Simonsen’s criteria after three and six months. Simonsen’s criteria of sealant retention was used due to simplicity, convenience, good reliability and high validity (8). Several authors have used Simonsen’s criteria of sealant retention supporting its high validity and reproducibility (22-24).

In the present study, completely retained sealants were seen more in Acid Etch with Air Abrasion pretreatment group compared to Acid Etching alone but the difference was not statistically significant after three and six months follow up. This may be due to enamel surface morphology after preparation with air abrasion being similar to acid etching. Yazici *et al.* compared acid etch and acid etch with air abrasion pretreatment techniques and found no significant difference in sealant retention in the two techniques at six months but found the difference to be statistically significant after nine and twelve months (16). Kanellis *et al.* compared acid etching versus air abrasion and obtained similar sealant retention rates on occlusal surfaces evaluated after six months (25). They suggested use of air abrasion prior to acid etching may result in increased sealant retention.

Conflicting results have been seen in the various *in vitro* studies which assessed the comparison between the two techniques. Some studies found no significant difference between air abrasion combined with acid etching compared to acid etching alone but concluded air abrasion when used alone led to least sealant retention (7,26). Knobloch *et al.* compared the effect of air abrasion, acid etching and the combination of both procedures on shear bond strength of sealant to primary enamel and found the combination of air abrasion and acid etching resulted in superior bond strength (27). They suggested that the increased surface area and contours created at macroscopic level by air abrasion, along with the micropores created by acid etching accounted for increased bond strength when both these techniques are used together.

Another comparison studied was sealant retention in primary molars versus permanent molars. According to Feigal *et al.*, no significant difference was reported in retention of sealants in primary and permanent molars while Doyle *et al.* found sealants to be more effective in permanent molars (28,29). In the present study, sealant retention was more in permanent teeth compared to primary teeth but the difference was not statistically significant. This may be due to strict isolation under rubber dam for both tooth types and adherence to proper technique of sealant placement.

Sealant retention according to tooth arch was also studied comparing maxillary molars with mandibular molars. Few studies reported superior sealant retention in maxillary teeth compared to mandibular teeth (30,31). Another study found no difference in sealant retention in maxillary and mandibular teeth (32). In the present study, maxillary molars retained sealants better than the mandibular molars which was highly significant after both three and six months (*p* value <0.05). This may be due to longer occlusal fissures and grooves in mandibular molars compared to maxillary molars which may limit the retention of sealants as more sealant gets exposed to the oral cavity (33). The maxillary molars have more roots than the mandibular components and therefore have more surface area to dissipate loads in the fine trabecular bone located in this region of the mouth. The greater dissipation of occlusal forces in maxillary molars compared to mandibular molars may have also led to better sealant retention in maxillary teeth as seen in this study.

## Conclusions

Within the limitations of this study, it may be concluded that:

1. Combining air abrasion pretreatment with subsequent acid etching did not result in statistically significant difference in sealant retention compared to acid etching alone in both primary and permanent molars after 3 and 6 months follow up.

2. Completely retained sealants were found to be more in the group where air abrasion pretreatment was combined with acid etching but the difference was not statistically significant.

3. Retention of sealants in permanent molars was superior to primary molars but the difference was not statistically significant.

4. Sealant retention was influenced according to tooth location with maxillary molars showing better retention compared to mandibular molars which was statistically significant.

It can be suggested through the present study that an additional air abrasion pretreatment step can be avoided in pediatric patients and the procedure can be completed faster with better behavior management. Further research is required at microscopic level to understand the bonding of sealant to tooth surface when air abrasion pretreatment is used with conventional acid etching. Long term clinical trials with other enamel pretreatment techniques should be conducted to find out the best method to improve retention of pit and fissure sealants. As studies on sealant retention in primary teeth are few, more research should be done comparing primary and permanent teeth to find the impact of morphological (eg: shallow pits and fissures in primary molars) and histololgical differences (eg: prismless enamel in primary teeth) between the two types of dentition on sealant retention.

## References

[1] Simonsen RJ (2002). Pit and fissure sealant: Review of the literature. Pediatr Dent.

[2] Wendt LK, Koch G, Birkhed D (2001). On the retention and effectiveness of fissure sealant in permanent molars after 15-20 years: a cohort study. Community Dent Oral Epidemiol.

[3] Ahovuo-Saloranta A, Hiiri A, Nordblad A, Makela M, Worthington HV (2008). Pit and fissure sealants for preventing dental decay in the permanent teeth of children and adolescents. Cochrane Database Syst Rev.

[4] Feigal RJ (1998). Sealants and preventive restorations: review of effectiveness and clinical changes for improvement. Pediatr Dent.

[5] Beauchamp J, Caufield PW, Crall JJ, Donly K, Feigal R, Gooch B (2008). Evidence-based clinical recommendations for the use of pit-and-fissure sealants. A report of the American Dental Association Council on Scientific Affairs. J Am Dent Assoc.

[6] Garcia-Godoy F, de Araujo FB (1994). Enhancement of fissure sealant penetration and adaptation: The enameloplasty technique. J Clin Pediatr Dent.

[7] Guirguis R, Lee J, Conry J (1999). Microleakage of restorations prepared with air abrasion. Pediatr Dent.

[8] Simonsen RJ (1991). Retention and Effectiveness of Dental Sealant After 15 Years. J Am Dent Assoc.

[9] Duangthip D, Lussi A (2003). Effects of fissure cleaning methods, drying agents, and fissure morphology on microleakage and penetration ability of sealants in vitro. Pediatr Dent.

[10] Gwinnett AJ, Buonocore MG (1965). Adhesives and caries prevention. Br Dent J.

[11] Crowe RA Jr (1971). An in vitro study of a fissure sealant. J La Dent Assoc.

[12] Rock W, Bradnock G (1981). Effect of operator variability and patient age on the retention of fissure sealant resin: 3-year results. Commun Dent Oral Epidemiol.

[13] Anson R, Full C, Wei S (1982). Retention of pit and fissure sealants placed in a dental school pedodontic clinic: a retrospective study. Pediatr Dent.

[14] Macek MD, Beltrán-Aguilar ED, Lockwood SA, Malvitz DM (2003). Updated comparison of the caries susceptibility of various morphological types of permanent teeth. J Public Health Dent.

[15] Waggoner WF, Siegal M (1996). Pit and fissure sealant application:updating the technique. J Am Dent Assoc.

[16] Yazici A, Kiremitci A, Celik C, Ozgunaltay G, Dayangac B (2006). A two-year clinical evaluation of pit and fissure sealants placed with and without air abrasion pretreatment in teenagers. J Am Dent Assoc.

[17] Silverstone LM (1974). Fissure sealants: Laboratory studies. Caries Res.

[18] Tandon S, Kumari R, Udupa S (1988). The effect of etch-time on the bond strength of a sealant and on the etch-pattern in primary and permanent enamel: an evaluation. ASDC J Dent Child.

[19] Laurell KA, Hess JA (1995). Scanning electron micrographic effects of air-abrasion cavity preparation on human enamel and dentin. Quintessence Int.

[20] Hegde VS, Khatavkar RA (2010). A new dimension to conservative dentistry: Air abrasion. J Conserv Dent.

[21] Horowitz HS, Heifetz SB, McCune RJ (1974). The effectiveness of an adhesive sealant in preventing occlusal caries: findings after two years in Kalispell, Mont. J Am Dent Assoc.

[22] Garrocho-Rangel A, Lozano-Vázquez C, Butron-Tellez-Giron C, Escobar-García D, Ruíz-Rodriguez S, Pozos-Guillen A (2015). In vitro assessment of retention and microleakage in pit and fissure sealants following enamel pre-etching with sodium hypochlorite deproteinisation. Eur J Paediatr Dent.

[23] Reddy VR, Chowdhary N, Mukunda KS, Kiran NK, Kavyarani BS, Pradeep MC (2015). Retention of resin-based filled and unfilled pit and fissure sealants: A comparative clinical study. Contemp Clin Dent.

[24] Subramaniam P, Konde S, Mandanna DK (2008). Retention of a resin-based sealant and a glass ionomer used as a fissure sealant: a comparative clinical study. J Indian Soc Pedod Prev Dent.

[25] Kanellis MJ, Warren JJ, Levy SM (2000). A comparison of sealant placement techniques and 12-month retention rates. J Public Health Dent.

[26] Borsatto MC, Corona SA, Dibb RG, Ramos RP, Pecora JD (2001). Microleakage of a resin sealant after acid etching, Er:YAG laser irradiation and air-abrasion of pits and fissures. J Clin Laser Med Surg.

[27] Knobloch LA, Meyer T, Kerby RE, Johnston W (2005). Microleakage and bond strength of sealant to primary enamel comparing air abrasion and acid etch techniques. Pediatr Dent.

[28] Feigal RJ (2002). The use of pit and fissure sealants. Pediatr Dent.

[29] Doyle W, Brose J (1978). A Five-Year Study on the Longevity of Fissure Sealants. ASDC J Dent Child.

[30] Burt BA, Berman DS, Gelbier S, Silverstone LM (1975). Retention of a fissure sealant six months after application. Br Dent J.

[31] Whitehurst V, Soni NN (1976). Adhesive sealant clinical trial: results eighteen months after one application. J Prev Dent.

[32] McCune RJ, Bojanini J, Abodeely RA (1979). Effectiveness of a pit and fissure sealant in the prevention of caries: three year clinical results. J Am Dent Assoc.

[33] Baca P, Bravo M, Baca AP, Jimenez A, Gonzalez-Rodríguez MP (2007). Retention of three fissure sealants and a dentin bonding system used as fissure sealant in caries prevention: 12-month follow-up results. Med Oral Patol Oral Cir Bucal.

